# Population-based cohort study: proton pump inhibitor use during pregnancy in Sweden and the risk of maternal and neonatal adverse events

**DOI:** 10.1186/s12916-022-02673-x

**Published:** 2022-12-20

**Authors:** Esmee M. Breddels, Johanna Simin, Romina Fornes, Helene Lilja Engstrand, Lars Engstrand, Robin Bruyndonckx, Nele Brusselaers

**Affiliations:** 1 I-BioStat, Data Science Institute, Hasselt University, Hasselt, Belgium; 2 Centre for Translational Microbiome Research, Department of Microbiology Tumor and Cell Biology, Karolinska Institutet, Biomedicum A8, Solnavägen 9, 17165 Stockholm, Sweden; 3 Department of Women’s and Children’s Health, Karolinska University Hospital, Stockholm, Sweden; 4 Global Health Institute, Antwerp University, Antwerp, Belgium

**Keywords:** Proton pump inhibitors, Pregnancy, Maternal and neonatal health, Logistic regression

## Abstract

**Background:**

Approximately half of all women suffer from heartburn at some stage during pregnancy. The most effective treatment is proton pump inhibitors, but the safety of use during pregnancy cannot be guaranteed. This study aimed to elucidate the effect of proton pump inhibitors on the risk of pre-eclampsia, gestational diabetes mellitus, preterm birth, an Apgar score at 5 min below 7, and a child being small or large for its gestational age.

**Methods:**

This Swedish population-based study included 1,089,514 live singleton deliveries between July 2006 and December 2016 in Sweden. Multiple logistic regression was used to model the outcomes as a function of the covariates. Results were presented as odds ratios with 95% confidence intervals.

**Results:**

In 1.4% of all pregnancies, the mother used proton pump inhibitors in the period from 3 months before the last menstrual period up to delivery. The use of proton pump inhibitors was associated with higher odds of pre-eclampsia (odds ratio = 1.19, 1.10–1.29), gestational diabetes mellitus (odds ratio = 1.29, 1.16–1.43), preterm birth (odds ratio = 1.23, 1.14–1.32), and small for gestational age (odds ratio = 1.27, 1.16–1.40) and lower odds of large for gestational age (odds ratio = 0.84, 0.77–0.91). No significant association was found with a low Apgar score 5 min after birth.

**Conclusions:**

Proton pump inhibitor use was associated with a higher risk of pre-eclampsia, gestational diabetes, preterm birth, and being born small for gestational age.

**Supplementary information:**

The online version contains supplementary material available at 10.1186/s12916-022-02673-x.

## Background

Proton pump inhibitors (PPIs) are the most effective treatment of heartburn [1], but contra-indicated during pregnancy. Yet PPIs are still prescribed in approximately 1% of all pregnancies according to our recent meta-analysis [2], and also available over-the-counter in several countries including Sweden [3, 4]. Maternal PPI use might affect the child via different mechanisms. PPIs have been shown to cross the placenta [1] and prenatal exposure to PPIs is seemingly associated with an increased risk of developing childhood asthma [5]. In addition, the initial gut colonization is highly influenced by the maternal microbiome (vaginal and fecal) [6, 7], and the maternal microbiome seems to play an important role in the onset of pregnancy complications [8–10]. In turn, PPI use has been associated with important changes in the gut microbiome that appear to be more prominent than those related to antibiotic use [11], also in infants as shown in our small pilot study [12]. The Food and Drug Administration (FDA) used to classify (up till 2015) most PPIs as category B drugs (“No risk in animal studies”), except for omeprazole which was categorized as type C (“Risk cannot be ruled out”) [13, 14]. Current recommendations are that omeprazole is not recommended during breastfeeding and that it “should be used during pregnancy only if the benefit outweighs the risk to the fetus” [15]. There were no reports of teratogenicity, and PPI use was not associated with major adverse pregnancy outcomes or birth defects [16–19], but was related to a lower birth weight [16, 17] and an increased risk of pre-eclampsia [20]. However, our recent meta-analysis reported an increased risk of congenital malformations associated with PPI use during pregnancy [2]. Little research is done concerning the effect of PPIs on less severe health risks that might have long-term implications for the mother and her offspring including maternal complications (gestational diabetes, pre-eclampsia), preterm birth, and small or large for gestational age. In our previous meta-analysis, we did find a handful of studies addressing neonatal adverse events, yet none of the pooled analyses (beyond congenital malformations) reached statistical significance, which may be due to power issues and low prevalences of exposure to PPIs [2]. This highlights the need to determine the effect of PPIs on the pregnant woman and her developing child.

This large nationwide population-based Swedish cohort study aimed to investigate the relation between the use of PPIs shortly before and during pregnancy on the risk of maternal and neonatal health complications.

## Methods

The study was performed using a Swedish cohort including all live singleton births delivered between July 2006 and December 2016, and the terminology “women” and “mother” were defined based on their biological sex and pregnancy status, not their gender identity. The cohort was created by linking information from four high-quality nationwide Swedish health data registries maintained by the National Board of Health and Welfare (Socialstyrelsen), as described earlier [21, 22]: the Medical Birth Registry [23–25] (established in 1973), the Prescribed Drug Registry [26] (established in July 2005), the Patient Registry (in- and outpatient care) [27, 28], and the Causes of Death Registry (since 1952). Information was linked through the unique Swedish personal identification number [29]. The study was approved by the Regional Ethics Committee of Stockholm (2017/2423–31), without the need for informed consent because of the registry-based nature of the data.

### Outcomes

The maternal outcomes were pre-eclampsia, characterized by hypertension (systolic blood pressure > 140 mmHg and/or diastolic blood pressure > 90 mmHg) combined with proteinuria (24-h urine protein level > 300 mg), and gestational diabetes mellitus (GDM), defined as any degree of glucose intolerance with the onset during pregnancy (Additional file 1: Table A1) [30]. The neonatal outcomes included preterm birth (birth < 37 weeks of gestation), an Apgar score 5 min after birth (AS_5min_) < 7, and small (SGA) and large for gestational age (LGA) based on birth weight in the 10th or 90th percentile based on gestational age, defined as birthweight below the 10th and above the 90th population percentile, respectively. [30, 31]

### Study exposure

The drugs in the Prescribed Drug Registry are classified according to the Anatomical Therapeutic Chemical (ATC) Classification System and the duration of use is expressed in defined daily doses (DDDs) per package. The exposure was prescribed PPI (ATC-code: A02BC) in the period ranging from 3 months before the last menstrual period (LMP) up to the delivery date. Women filling at least two prescriptions during the study period were considered users since lower compliance is expected for those with only a single prescription. [32]

### Covariates

Potential confounders included maternal characteristics (age at delivery, body mass index (BMI), tobacco consumption (smoking or moist snuff use), other prescribed drug use, comorbidities), pregnancy, and obstetric characteristics (Additional file 1: Table A1). Missingness in BMI was adjusted for by creating an additional dummy variable. The use of prescription drugs was split into histamine-2 receptor antagonist (H_2_RA) use and other drugs (NSAIDs, low-dose aspirin, and antibiotics). The use of H_2_RA was separated because it is prescribed for similar indications as PPIs. Maternal comorbidities were identified according to their ICD-10 codes or by the prescription of associated drugs (Additional file 1: Table A1). The comorbidities included hypertension, GDM, diabetes mellitus (type 1 and type 2), and hypo- and hyperthyroidism.

Pregnancy and obstetric variables, of which some are also outcomes, expected to be associated with at least one outcome were pre-eclampsia, mode of delivery (cesarean section or vaginal delivery), preterm birth, neonatal birthweight (SGA, average for gestational age (AGA) or LGA), parity, time in months since previous delivery, and whether the outcome was present in a previous pregnancy.

### Statistical analysis

The effect of PPIs was assessed by comparing PPI users with non-users. Multiple logistic regression models were used to evaluate the association between the exposure and the odds that the outcome occurred, corrected for covariates, and presented as odds ratios (OR) with 95% confidence intervals (CI).

Models were built independently for each outcome, based on the purposeful selection method described by Hosmer et al. [33]. For more detail, see Additional file 2: Additional Methods. [33–37]

All final models included PPI use, irrespective of whether it was significant, because it was the exposure of interest. The model was concluded as the final model after assessing the adequacy and fit of the model. The cohort contained women with one or more pregnancies resulting in live birth. Generalized Estimating Equations (GEE) were used to take the correlation between siblings into account [38]^.^

All analyses were performed in R version 3.6.1 [39–42]. Observations with missing information on one of the outcomes (*n* = 6105) were removed from all analyses. All covariates were categorical and, if necessary, included a separate category for missing values.

### Sensitivity analysis (only firstborns)

Multiple linear logistic regression was performed for each outcome, including only the firstborn children. Covariates included all before-mentioned maternal characteristics. Pregnancy and obstetric characteristics included were pre-eclampsia, mode of delivery, preterm birth, and neonatal birthweight, excluding covariates considering multiple pregnancies.

### Use of PPIs at different timepoints (trimesters)

To determine if the use of PPIs had a different effect depending on the timing of the prescription, the exposure was split into four time periods. The first period ranged from 3 months before up to the last menstrual period (LMP). The time between the LMP and the delivery was divided into trimesters. The first trimester lasted until 97 days after LMP, the second starting at 98 days until 202 days of gestation, and the third trimester ranged from 203 days of gestation to delivery.

### Dose–response association

The dose–response association between PPI use and the outcomes was assessed in two models. First, PPI use was replaced by a categorical variable describing the number of prescriptions (≤ 1, 2, 3, ≥ 4). Second, PPI use was described by a categorical variable based on the quantiles of the DDD presented as the number of weeks.

## Results

### Descriptive characteristics

The study included 1,089,514 live singleton births delivered between July 2006 and December 2016 in Sweden. PPIs were used by 1.4% (*n* = 14,787) of the women 3 months before or during gestation (Table 1). Overall, PPI users were older and had a higher BMI at the start of pregnancy. Among PPI users, the reported obesity was almost double (21.6%) compared to PPI non-users (11.7%). Users had a higher frequency of comorbidities (11.9% of users and 5.8% of non-users) and/or exposure to other drugs than PPIs (53.6% and 31.8% respectively). The use of PPIs was more prevalent among the third or higher pregnancy of the mother.

**Table 1:** Distribution of maternal and obstetric characteristics among all singleton pregnancies resulting in livebirth in Sweden, by exposure to proton pump inhibitors (PPIs)

	Total		PPI users		PPI non-users	
	*N*	%	*N*	%	*N*	%
Total	1,089,515	100.00%	14,787	1.40%	1,074,728	98.60%
Maternal age (years) at delivery						
≤ 25	206,620	19.00%	2123	14.40%	204,497	19.00%
25–30	353,028	32.40%	4321	29.20%	348,707	32.40%
30–35	347,795	31.90%	4850	32.80%	342,945	31.90%
> 35	182,072	16.70%	3493	23.60%	178,579	16.60%
Body mass index(kg/m^2^) at enrolment						
< 20	105,145	9.70%	1122	7.60%	104,023	9.70%
20–25	526,129	48.30%	5548	37.50%	520,581	48.40%
25–30	256,024	23.50%	3957	26.80%	252,067	23.50%
≥ 30	129,030	11.80%	3190	21.60%	125,840	11.70%
Missing	73,187	6.70%	970	6.60%	72,217	6.70%
Tobacco consumption						
Yes	75,600	6.90%	1335	9.00%	74,265	6.90%
No	1,013,915	93.10%	13,452	91.00%	1,000,463	93.10%
Comorbidities*						
Yes	64,076	5.90%	1755	11.90%	62,321	5.80%
No	1,025,439	94.10%	13,032	88.10%	1,012,407	94.20%
Exposure to H_2_ receptor antagonists						
Yes	2321	0.20%	320	2.20%	2001	0.20%
No	1,087,194	99.80%	14,467	97.80%	1,072,727	99.80%
Exposure to other drugs**						
Yes	349,252	32.10%	7929	53.60%	341,323	31.80%
No	740,263	67.90%	6858	46.40%	733,405	68.20%
Assisted reproduction						
Yes	31,799	2.90%	574	3.90%	31,225	2.90%
No	1,057,716	97.10%	14,213	96.10%	1,043,503	97.10%
Mode of delivery						
Vaginal	906,006	83.20%	11,275	76.20%	894,731	83.30%
Cesarean section	183,509	16.80%	3512	23.80%	179,997	16.70%
Parity						
1	481,573	44.20%	6140	41.50%	475,433	44.20%
2	400,762	36.80%	4607	31.20%	396,155	36.90%
≥ 3	207,180	19.00%	4040	27.30%	203,140	18.90%
Time interval between pregnancies (months)						
< 18	141,036	12.90%	1751	11.80%	139,285	13.00%
18–23	63,910	5.90%	737	5.00%	63,173	5.90%
> 23	164,468	15.10%	2676	18.10%	161,792	15.10%
0	720,101	66.10%	9623	65.10%	710,478	66.10%

^*^Comorbidities defined as at least one diagnosis of hypertension, diabetes mellitus, and hypo- or hyperthyroidism

^**^Other drugs defined as at least one prescription of non-steroid anti-inflammatory drugs (NSAIDs), low-dose aspirin, or antibiotics 3 months before or during gestation

Among PPI users, 4.9% developed pre-eclampsia and 3.2% GDM, whereas these complications occurred in respectively 3.3% and 1.4% of pregnancies in non-users (Table 2). Children were born preterm in 6.5% and had a low AS_5min_ in 1.6% of the deliveries among PPI users and respectively 4.6% and 1.2% in non-users. The birthweight of the child was SGA or LGA in respectively 3.2% and 3.9% among users and 2.3% and 3.5% among non-users.

**Table 2:** Distribution of the outcomes among all singleton pregnancies resulting in livebirth in Sweden, by proton pump inhibitor (PPI) exposure

	Total		PPI users		PPI non-users	
	*N*	%	*N*	%	*N*	%
Total	1,089,515	100.00%	14,787	1.40%	1,074,728	98.60%
Pre-eclampsia						
Yes	35,791	3.30%	723	4.90%	35,068	3.30%
No	1,053,724	96.70%	14,064	95.10%	1,039,660	96.70%
Gestational diabetes						
Yes	15,958	1.50%	475	3.20%	15,483	1.40%
No	1,073,557	98.50%	14,312	96.80%	1,059,245	98.60%
Preterm birth						
Yes	50,765	4.70%	966	6.50%	49,799	4.60%
No	1,038,750	95.30%	13,821	93.50%	1,024,929	95.40%
Low Apgar score						
Yes	12,834	1.20%	239	1.60%	12,595	1.20%
No	1,076,681	98.80%	14,548	98.40%	1,062,133	98.80%
Birthweight for gestational age						
Small	24,690	2.30%	472	3.20%	24,218	2.30%
Average	1,027,099	94.20%	13,737	92.90%	1,013,362	94.30%
Large	37,726	3.50%	578	3.90%	37,148	3.50%

^*^Comorbidities defined as at least one diagnosis of hypertension, diabetes mellitus, and hypo- or hyperthyroidism

^**^Other drugs defined as at least one prescription of non-steroid anti-inflammatory drugs (NSAIDs), low-dose aspirin, or antibiotics 3 months before or during gestation

### Other risk factors

Large increasing effects on at least one of the outcomes were shown for BMI, smoking, comorbidities, and mode of delivery (all time periods combined). Women with a higher BMI or comorbidities had higher odds of pre-eclampsia and gestational diabetes and giving birth to a LGA child (Additional file 3: Table A2). Smoking was associated with over twofold odds of the child being SGA (OR = 2.03, 95% CI 1.95–2.11) and a c-section affected the odds of a low AS_5min_ (OR = 3.33, 95% CI 3.21–3.46). Among women diagnosed with pre-eclampsia during the pregnancy, the odds of preterm birth (OR = 4.74, 95% CI 4.60–4.89) and SGA (OR = 4.46, 95% CI 4.30–4.63) were increased. If the women had a history of pre-eclampsia or GDM, they had an OR of respectively 5.20 (4.76–5.68) and 8.98 (7.92–10.17) to have the outcome again. The odds of delivering preterm and a low AS_5min_ were slightly higher if it occurred during a previous pregnancy of the women. The child had higher odds of being SGA or LGA if their sibling was SGA (OR = 8.16, 95% CI 7.55–8.81) or LGA (OR = 11.05, 95% CI 10.52–11.61), respectively.

### Use of PPIs and the risk of maternal and neonatal health effects

The use of PPIs increased the odds of the mother developing pre-eclampsia and GDM respectively with 19 and 29% compared to non-users (OR = 1.19, 95% CI 1.10–1.29 and OR = 1.29, 95% CI 1.16–1.43) (Table 2). The neonate of a PPI user had an OR of 1.23 (95% CI 1.14–1.32) to be born preterm compared to non-users. The odds of a low AS_5min_ was not significantly affected by PPI use of the mother. The use of PPIs increased the odds of a SGA child (OR = 1.27, 95% CI 1.16–1.40), whereas it was associated with a decrease in the odds for LGA (OR = 0.84, 95% CI 0.77–0.91).

### Subset analysis (only firstborns)

Including only the firstborn children in the data resulted in similar associations as the analysis including all live births (Additional file 4: Table A3).

### Use of PPIs at different timepoints (trimesters)

The odds of developing pre-eclampsia increased when PPIs were used during the second (OR = 1.38, 95% CI 1.16–1.65) or third (OR = 1.45, 95% CI 1.20–1.76) trimester of pregnancy (Table 3). If a woman had PPIs prescribed during the second and third trimester, her odds of pre-eclampsia was 1.29 (95% CI 1.12–1.50). The use of PPIs only in the 3 months before the LMP was significantly associated with and increased odds of giving birth to a child preterm (OR = 1.26, 95% CI 1.12–1.42) and of the child being SGA (OR = 1.42, 95% CI 1.11–1.82). PPI use only in the first trimester gave a slight increase in the odds of preterm birth (OR = 1.16, 95% CI 1.02–1.30) and SGA (OR = 1.25, 95% CI 1.00–1.57). The odds of preterm birth were mainly affected by the use of PPIs during the second trimester (OR = 1.58, 95% CI 1.40–1.78). In contrast, PPI use in the third trimester reduced the odds of preterm birth (OR = 0.54, 95% CI 0.47–0.61).

**Table 3:** Associations between PPI exposure at different timepoints during pregnancy and maternal and neonatal health outcomes expressed as odds ratios (OR) with 95% confidence interval (CI) obtained by multiple logistic regression including all live births

PPI use	Pre-eclampsia	GDM	Preterm	AS_5min_ < 7	SGA	LGA
Overall	1.19 (1.10–1.29)	1.29 (1.16–1.43)	1.23 (1.14–1.32)	1.07 (0.93–1.22)	1.27 (1.16–1.40)	0.84 (0.77–0.91)
Before pregnancy (3 months)	0.92 (0.79–1.06)	1.07 (0.88–1.30)	1.26 (1.12–1.42)	1.13 (0.89–1.44)	1.42 (1.11–1.82)	0.89 (0.71–1.12)
First trimester	0.91 (0.78–1.05)	1.20 (0.99–1.46)	1.16 (1.02–1.30)	1.06 (0.85–1.34)	1.25 (1.00–1.57)	0.81 (0.66–1.00)
Second trimester	1.38 (1.16–1.65)	0.97 (0.80–1.18)	1.58 (1.40–1.78)	1.15 (0.93–1.43)	1.06 (0.84–1.33)	0.87 (0.70–1.08)
Third trimester	1.45 (1.20–1.76)	1.20 (1.00–1.45)	0.54 (0.47–0.61)	0.84 (0.66–1.06)	0.85 (0.63–1.13)	0.79 (0.62–1.01)
Before/first trimester*					0.62 (0.42–0.90)	1.28 (0.90–1.80)
Second/third trimester**	0.64 (0.49–0.85)				1.46 (0.98–2.16)	1.41 (1.00–2.00)

^*^Interaction term for PPI use 3 months before pregnancy and during the first trimester

^**^Interaction term for PPI use in second and third trimesters of pregnancy

Abbreviations: *AS*_*5min*_, Apgar score 5 min after birth; *GDM*, gestational diabetes; *LGA*, large for gestational age; *PPI*, proton pump inhibitor; *SGA*, small for gestational age

### Dose–response association

Women with 2 (OR = 1.21, 95% CI 1.09–1.36) or 3 (OR = 1.30, 95% CI 1.11–1.52) prescriptions had higher odds of pre-eclampsia compared to non-users (Table 4). An increased odds of GDM was associated with any number of PPI prescriptions, yet without large differences in the odds of GDM between the different categories. All prescriptions increased the odds of preterm birth, with 3 prescriptions (OR = 1.56, 95% CI 1.37–1.78) having a larger effect compared to 2 (OR = 1.11, 95% CI 1.01–1.23) or at least 4 (OR = 1.19, 95% CI 1.03–1.36) prescriptions. The odds of SGA was higher in women with 2 (OR = 1.31, 95% CI 1.14–1.49) or at least 4 (OR = 1.28, 95% CI 1.05–1.56) prescriptions compared to non-users, and the effect was not higher in women with 4 or more prescriptions than in women with 2 prescriptions. Filling of 2 prescriptions was significantly associated with lower odds of LGA (OR = 0.81, 95% CI 0.71–0.92).

**Table 4:** Association between the use of PPI and maternal and neonatal outcomes presented as the odds ratio of having the outcome compared to non-users

PPI use	Pre-eclampsia	GDM	Preterm	AS_5min_ < 7	SGA	LGA
Number of prescriptions						
2	1.21 (1.09–1.36)	1.24 (1.07–1.44)	1.11 (1.01–1.23)	1.00 (0.83–1.21)	1.31 (1.14–1.49)	0.81 (0.71–0.92)
3	1.30 (1.11–1.52)	1.35 (1.10–1.67)	1.56 (1.37–1.78)	1.07 (0.81–1.40)	1.19 (0.97–1.47)	0.85 (0.71–1.03)
≥ 4	1.05 (0.89–1.24)	1.32 (1.10–1.59)	1.19 (1.03–1.36)	1.19 (0.93–1.53)	1.28 (1.05–1.56)	0.87 (0.74–1.02)
Number of weeks						
0–12	1.34 (1.16–1.54)	1.38 (1.14–1.68)	1.06 (0.93–1.21)	1.02 (0.79–1.32)	1.14 (0.94–1.37)	0.82 (0.69–0.97)
12–20	1.19 (1.00–1.42)	1.41 (1.14–1.75)	1.29 (1.12–1.49)	1.01 (0.75–1.37)	1.29 (1.05–1.60)	0.92 (0.77–1.11)
20–36	1.23 (1.05–1.43)	1.14 (0.92–1.40)	1.16 (1.01–1.34)	0.96 (0.73–1.28)	1.45 (1.20–1.74)	0.79 (0.66–0.95)
> 36	1.01 (0.86–1.18)	1.26 (1.04–1.52)	1.45 (1.27–1.65)	1.24 (0.98–1.57)	1.25 (1.03–1.52)	0.83 (0.70–0.98)

Abbreviations: *AS*_*5min*_, Apgar score 5 min after birth; *DDD*, defined daily doses; *GDM*, gestational diabetes; *LGA*, large for gestational age; *PPI*, proton pump inhibitor; *SGA*, small for gestational age

A DDD up to and including 36 weeks increased the odds of developing pre-eclampsia compared to non-users. No significant difference was found between the different categories. DDDs up to 20 weeks and over 36 weeks were associated with higher odds of GDM, without differences across the categories. No association was found between a DDD of 20–36 weeks and the odds of GDM. An increased odds of preterm birth and SGA was associated with PPI use longer than 12 weeks, but no large differences between the DDD categories. A slight decrease in the odds of LGA was associated with a DDD of 0–12 (OR = 0.82, 95% CI 0.69–0.97), 20–36 (OR = 0.79, 95% CI 0.66–0.95), and more than 36 (OR = 0.83, 95% CI 0.70–0.98) weeks.

## Discussion

In this large Swedish population-based study, PPI use shortly before and during pregnancy was associated with a higher probability of pre-eclampsia, GDM, preterm birth, and being born SGA. Analysis of only the firstborn child of a mother yielded similar results. Differences in the outcomes were seen by the different exposure periods based on all pregnancies. PPI use in the period ranging from three months before LMP until the end of the first trimester was associated with increased odds of preterm birth and SGA. Similarly, the odds of preterm birth were also higher when PPIs were used during the second trimester. PPI use in the second and third trimesters of pregnancy was associated with higher odds of pre-eclampsia. No evidence for a dose–response relation between PPI use and any of the outcomes was found.

Our results are consistent with previous studies relating PPI use to a higher (or not reduced) risk of pre-eclampsia [20, 43, 44] and low birth weight [16, 17]. Contradicting to our results, other observational studies have reported no significant relation between PPI use and low birthweight and/or preterm birth [17, 19]. However, compared to this large nationwide study, both studies had a much lower number of observations available and mainly focused on major anomalies. To our knowledge, there are no randomized clinical trials investigating the safety of maternal PPI use, regarding maternal and neonatal adverse events [2, 45, 46]. We also question if it is still ethically defendable to conduct these on PPI use during pregnancy with the accumulating safety concerns based on association studies, and our increasing understanding of drug interactions and the microbiome [2, 5, 12, 47, 48].

The maternal and neonatal adverse events investigated can affect short- and long-term health of both the mother and the child. Pre-eclampsia is a cause of worldwide maternal and perinatal morbidity and mortality [49]. GDM has previously been associated with an increased frequency of maternal hypertensive disorders and an increased risk of type 2 diabetes after pregnancy. GDM has been associated with the child having a higher odds of developing obesity, glucose intolerance, and diabetes in late adolescence and young adulthood [50]. Preterm birth is a major cause of neonatal and infant morbidity. Children that are too small or too light at birth, have a higher risk of hypertension, obesity, and diabetes mellitus type 2 later in life [51] and a lower quality of life when young adults [52]. We do acknowledge that causality cannot be established and that unknown confounders may still affect the results. Although we did adjust for BMI, we did see that obesity was more prevalent among PPI users (21.6% vs. 11.7%) and was associated with higher odds of pre-eclampsia and gestational diabetes, preterm birth and large for gestational age, as previously described in the literature. [53, 54]

Despite being contra-indicated, 1.4% of pregnant women in this cohort were PPI users (excluding over-the-counter use). Many studies reporting PPI utilization during pregnancy report a prevalence below 2% [2, 5, 55, 56] with a few studies reporting up to 6% [43, 57, 58]. Although this is lower than reported utilization in non-pregnant adults [59–63], over-the-counter use is usually not included [64]. With 4 million pregnancies born in the European Union in 2020 alone, a 1% PPI prevalence equals 40,000 pregnancies per year [65]. As our results do support previous safety concerns [16–19], more awareness to potential consequences of PPI use during pregnancy seems warranted. To note, PPI use has been considered inappropriate in up to 70% of (non-pregnant) long-term users [66, 67].

The underlying mechanisms on how PPIs affect our health need further exploration; as well as safer (non-pharmaceutical) alternatives for treating of gastro-intestinal symptoms and discomfort during pregnancy. PPIs may still have a place for restricted indications during pregnancy, yet widespread and unsupervised over-the-counter use should be discouraged.

This study has several strengths including the large registry-based nature of the data and its high completeness, resulting in a large nationwide and population-based study with highly valid data on outcomes, exposure, and covariates.

Despite the high completeness of the registries and adjustments for confounders, confounding by indication could not be entirely ruled out. Nausea, vomiting (hyperemesis gravidarum), gastro-esophageal reflux, and/or peptic ulcers may be more common and/or severe among PPI users than non-users. Although these indications may increase the risk for adverse events, it remains unclear if PPI use can reduce the risk [68]. Reverse causation could be affecting the associations, particularly for third-trimester exposure, for which the effect could also be underestimated since not all deliveries reach the end of the third trimester.

In addition, information on the outcomes and some covariates was incomplete (< 1% overall). Observations with any of the outcome variables missing (0.006%) were removed. Information on the exposure was limited by the availability of PPIs over the counter and lack of confirmation whether the women actually used the drugs, although it is expected that most women will use prescribed drugs only (after advice from their midwife/clinician). We included only women with at least two dispensed prescriptions, indicating that they were utilizing the drugs. This, however, could lead to misclassification of women filling a single prescription taking the drug, or women who only took PPI over the counter. Another potential concern is that a woman with previously diagnosed diabetes might have been missed and/or misclassified as having GDM. Due to a lack of power (only 0.2% of this cohort used H2RA), it was not possible to assess the effect of H_2_RA use on the odds of an outcome. H_2_RA is prescribed for similar indications as PPIs and it is recommended only to prescribe PPIs if antiacids and H_2_RA do not sufficiently relieve symptoms [1, 13]. The registries did not provide data on potential confounders such as whether the mothers took tocolytics to suppress preterm delivery, chronic hypertension, and hyperemesis. We also lacked information on ethnicity and socio-economic status. Nonetheless, our results were adjusted for important factors including diabetes mellitus, gestational hypertension, and hypo- and hyperthyroidism. The current analysis only included whether the outcome of interest was present in a previous pregnancy, but not if any of the other outcomes was. Others reported on a previous LGA child increasing the risk of GDM [69] and previous SGA the risk of preterm birth [70]. Furthermore, we would not expect socio-economic differences to have influenced our results significantly, because pregnancy-related health care in Sweden is highly standardized, equally accessible for the entire country, and free for the expecting mother. We chose to categorize exposure by trimester above time-varying exposures, since this is the most applicable to clinical antenatal practice (in particular since any antenatal PPI use is contra-indicated). We also do not have the exact period of exposure, since the duration of use is estimated based on the average use per package.

## Conclusions

Our large study suggests an increase in the risk of pre-eclampsia, GDM, preterm birth, and SGA associated to maternal PPI use during pregnancy. Therefore, we believe PPIs should be prescribed more cautiously and only be used under clinical supervision during pregnancy.

## Supplementary information

**Additional file 1: Table A1.** Description of the dependent and independent variables used and if appropriate the ICD-10 and ATC-codes used to identify the presence of the variable.

**Additional file 2.** Additional methods.

**Additional file 3: Table A2.** Associations between PPI exposure and maternal and neonatal health outcomes including all live births. Results were obtained by multiple logistic regression and expressed as odds ratios (OR) with 95% confidence interval (CI). Empty cells indicated the variable was not included in the final model for the outcome. Abbreviations: AGA, average for gestational age; AS5min, Apgar score 5 min after birth; BMI, body mass index; GDM, gestational diabetes; LGA, large for gestational age; NA, not available; PPI, proton pump inhibitors; SGA, small for gestational age.

**Additional file 4: Table A3.** Associations between PPI exposure and maternal and neonatal health outcomes including only firstborns. Results were obtained by multiple logistic regression and expressed as odds ratios (OR) with 95% confidence interval (CI). Empty cells indicated the variable was not included in the final model for the outcome. Abbreviations: AGA, average for gestational age; AS5min, Apgar score 5 min after birth; BMI, body mass index; GDM, gestational diabetes; LGA, large for gestational age; NA, not available; PPI, proton pump inhibitors; SGA, small for gestational age.

## Data Availability

Upon reasonable request and after required approvals from the Ethics Committee and National Board of Health and Welfare are obtained. Because the data belong to the National Board of Health and Welfare (Socialstyrelsen) and the detailed level of clinical information, it is not allowed to share these data publicly.

## Abbreviations

AGA: Appropriate for gestational age
AS: Apgar score
ATC: Anatomical therapeutical classification
CI: Confidence interval
DDD: Defined daily dose
FDA: Food and Drug Administration
GDM: Gestational diabetes mellitus
GEE: Generalized Estimating Equations
H2RA: Histamine-2 receptor antagonist
OR: Odds ratio
LGA: Large for gestational age
PPI: Proton pump inhibitor
SGA: Small for gestational age
