# Supplementary material for: Population-based cohort study: proton pump inhibitor use during pregnancy in Sweden and the risk of maternal and neonatal adverse events
Source: BMC Med. 2022 Dec 20;20:492. doi: 10.1186/s12916-022-02673-x (PMC9768950; doi:10.1186/s12916-022-02673-x)
Supplement: Supplementary file 1 — Additional file 1: Table A1. Description of the dependent and independent variables used and if appropriate the ICD-10 and ATC-codes used to identify the presence of the variable. [file 12916_2022_2673_MOESM1_ESM.docx]

ADDITIONAL FILE 1: Table A1: Description of the dependent and independent variables used and if appropriate the ICD-10 and ATC-codes used to identify the presence of the variable.

| Variable | Description | ICD10-codes | ATC-codes |
| --- | --- | --- | --- |
| **Outcomes** |  |  |  |
| Pre-eclampsia | Hypertension (systolic blood pressure > 140 mmHg and/or diastolic blood pressure > 90 mmHg) combined with proteinuria (24h urine protein level > 300 mg) | O14 |  |
| Gestational diabetes | Any degree of glucose intolerance with the onset during pregnancy | O24.0, O24.1, O24.4, E10, E11 | A10A |
| Preterm birth | Delivery before 37 completed weeks of gestation |  |  |
| Apgar score at 5 min | Score reflecting neonatal health 5 minutes after birth ( ≥7 is considered healthy) |  |  |
| Small for gestational age | Birthweight below 10^th^ percentile |  |  |
| Large for gestational age | Birthweight above 90^th^ percentile |  |  |
| Exposure |  |  |  |
| Proton Pump Inhibitors | At least 2 prescriptions filled during the study period |  | A02BC |
| **Maternal characteristics** |  |  |  |
| Age | Age of the mother at the time of delivery in years |  |  |
| Body mass index | Calculated as kg/m^2^ from the weight and height of the mother at the time of registration in prenatal care |  |  |
| Tobacco consumption | Reported at the time of registration in prenatal care |  |  |
| Comorbidities | Combines the presence of diabetes type 1 and 2, hypertension during pregnancy and hypo- and hyperthyroidism | O24.0, O24.1, O24.4, E10, E11, H03A, H03B | A10A |
| H_2_RA use | At least one prescription filled |  | A02BA |
| Other drug use | At least one prescription filled for NSAIDs or low dose aspirin or antibiotics |  | M01AE, B01AC06, N02BA01, J01 |
| **Obstetric characteristics** |  |  |  |
| Assisted reproduction | Assisted reproduction techniques were used to induce pregnancy |  |  |
| Mode of delivery | Delivery by caesarean section or vaginal |  |  |
| Parity | Number of times a woman has given birth to a fetus with a gestational age of 24 weeks or more |  |  |
| Pregnancy interval | Time in months between the delivery of a child and the last menstrual period of the next pregnancy |  |  |
