# Supplementary material for: Population-based cohort study: proton pump inhibitor use during pregnancy in Sweden and the risk of maternal and neonatal adverse events
Source: BMC Med. 2022 Dec 20;20:492. doi: 10.1186/s12916-022-02673-x (PMC9768950; doi:10.1186/s12916-022-02673-x)
Supplement: Supplementary file 2 — Additional file 2. Additional methods. [file 12916_2022_2673_MOESM2_ESM.docx]

# ADDITIONAL FILE 2: ADDITIONAL METHODS

Assumptions made by all models were: i) independence of the observations, ii) little or no multicollinearity among the independent variables, and iii) linear relation between the logit and the predictors. The cohort contained women with one or more pregnancies resulting in live birth. Generalized Estimating Equations (GEE) were used to take the correlation between siblings into account. The working correlation structure was determined by fitting intercept only models with different working correlations (exchangeable, independence, autoregressive and unstructured). The exchangeable working correlation was used unless any of the other models resulted in a much lower Quasi-likelihood under Independence Model Criterion (QIC) value.

Models were built independently for each outcome, based on the purposeful selection method described by Hosmer et al. First, univariable analysis was performed for each independent variable using Pearson χ^2^-test. Second, variables with a univariable significance at alpha 0.25 were included in the initial model. Third, the initial model was reduced by rounds of removing variables with the highest p-value and comparing the reduced with the larger model using Wald test (α=0.05). The process was repeated until all included variables were statistically significant. Next, variables excluded based on univariable analysis were added to the model, tested, and included if a significant association (α=0.05) was found. The model was extended by the addition of interaction terms that were clinically sensible and statistically significant (α=0.05). All final models included PPI use, irrespective whether it was significant, because it was the exposure of interest. The model was concluded as the final model after assessing the adequacy and fit of the model.

The fit of the models were assessed by Pearson χ^2^-statistic, deviance and Hosmer-Lemeshow test statistics, and by plots of standardized Pearson and deviance residuals against the predicted values. The variance inflation factor (VIF) was used to detect multicollinearity (VIF > 2.5). The parameter estimates and standard errors were obtained by GEE and inferences on the parameters were based on the Wald test. Parameters were presented and interpreted in terms of odds ratios (OR) with corresponding 95% confidence interval (CI).

The data was retrieved from a PostgreSQL database system through R using the package “RPostgreSQL”. Multinomial logistic regression was performed by using the “VGAM” package. Clustered binomial models were made using the packages “geepack”. Additional packages for summary measures and to support the before mentioned packages were used.
