# Supplementary material for: Population-based cohort study: proton pump inhibitor use during pregnancy in Sweden and the risk of maternal and neonatal adverse events
Source: BMC Med. 2022 Dec 20;20:492. doi: 10.1186/s12916-022-02673-x (PMC9768950; doi:10.1186/s12916-022-02673-x)
Supplement: Supplementary file 3 — Additional file 3: Table A2. Associations between PPI exposure and maternal and neonatal health outcomes including all live births. Results were obtained by multiple logistic regression and expressed as odds ratios (OR) with 95% confidence interval (CI). Empty cells indicated the variable was not included in the final model for the outcome. Abbreviations: AGA, average for gestational age; AS5min, Apgar score 5 min after birth; BMI, body mass index; GDM, gestational diabetes; LGA, large for gestational age; NA, not available; PPI, proton pump inhibitors; SGA, small for gestational age. [file 12916_2022_2673_MOESM3_ESM.docx]

ADDITIONAL FILE 3: Table A2: Associations between PPI exposure and maternal and neonatal health outcomes including all live births. Results were obtained by multiple logistic regression and expressed as odds ratios (OR) with 95% confidence interval (CI).

|  | | Pre-eclampsia | GDM | Preterm | AS_5min_ <7 | SGA | LGA |
| --- | --- | --- | --- | --- | --- | --- | --- |
| PPI | | 1.19 (1.10-1.29) | 1.29 (1.16-1.43) | 1.23 (1.14-1.32) | 1.07 (0.93-1.22) | 1.27 (1.16-1.40) | 0.84 (0.77-0.91) |
| Age | ≤25 | *ref* | *ref* | *ref* |  | *ref* | *ref* |
| 25-30 | | 0.99 (0.96-1.02) | 1.21 (1.15-1.29) | 0.98 (0.96-1.01) |  | 0.96 (0.92-0.99) | 1.05 (1.01-1.09) |
| 30-35 | | 1.08 (1.04-1.11) | 1.50 (1.41-1.59) | 0.98 (0.95-1.01) |  | 1.06 (1.02-1.10) | 1.08 (1.04-1.12) |
| >35 | | 1.38 (1.33-1.43) | 2.08 (1.95-2.21) | 1.12 (1.09-1.16) |  | 1.31 (1.25-1.37) | 1.10 (1.06-1.14) |
| BMI | Normal | *ref* | *ref* | *ref* | *ref* | *ref* | *ref* |
| Under | | 0.78 (0.74-0.82) | 0.75 (0.68-0.83) | 1.19 (1.16-1.23) | 0.90 (0.84-0.97) | 1.51 (1.45-1.57) | 0.46 (0.43-0.49) |
| Over | | 1.64 (1.60-1.69) | 2.11 (2.01-2.21) | 1.04 (1.01-1.06) | 1.20 (1.15-1.26) | 0.90 (0.87-0.93) | 1.82 (1.78-1.87) |
| Obese | | 2.97 (2.89-3.06) | 5.21 (4.98-5.45) | 1.11 (1.08-1.15) | 1.61 (1.53-1.69) | 0.91 (0.87-0.95) | 2.84 (2.76-2.93) |
| NA | | 1.45 (1.38-1.51) | 1.47 (1.36-1.58) | 1.81 (1.75-1.87) | 1.49 (1.40-1.58) | 1.13 (1.07-1.18) | 1.51 (1.45-1.58) |
| Tobacco consumption | | 0.82 (0.78-0.86) |  | 1.38 (1.33-1.42) |  | 2.03 (1.95-2.11) | 0.65 (0.62-0.68) |
| Comorbidities | | 2.01 (1.94-2.08) | 4.42 (4.23-4.63) | 1.73 (1.68-1.79) |  | 0.98 (0.93-1.04) | 2.45 (2.37-2.53) |
| Pre-eclampsia | |  | 1.58 (1.48-1.68) | 4.74 (4.60-4.89) |  | 4.46 (4.30-4.63) | 1.42 (1.35-1.50) |
| Other drugs | | 1.22 (1.20-1.25) | 1.17 (1.13-1.21) | 1.13 (1.11-1.16) | 1.08 (1.04-1.12) | 1.00 (0.98-1.03) | 1.11 (1.09-1.14) |
| Assisted reproduction | | 1.09 (1.03-1.15) | 1.41 (0.84-2.37) | 1.28 (1.22-1.34) |  | 1.03 (0.96-1.10) | 1.17 (1.10-1.25) |
| Mode of delivery | |  |  |  | 3.33 (3.21-3.46) |  |  |
| Preterm birth | |  |  |  | 5.47 (5.23-5.72) | 4.03 (3.89-4.16) | 1.35 (1.29-1.42) |
| Birthweight | AGA |  |  |  | *ref* |  |  |
| SGA | |  |  |  | 1.56 (1.45-1.68) |  |  |
| LGA | |  |  |  | 1.37 (1.26-1.48) |  |  |
| Parity | 1 |  |  |  |  |  |  |
| 2 | | 0.41 (0.39-0.42) | 1.09 (1.04-1.14) | 0.71 (0.70-0.73) | 0.58 (0.56-0.61) | 0.54 (0.52-0.56) | 2.28 (2.21-2.36) |
| >2 | | 0.35 (0.34-0.36) | 1.36 (1.30-1.43) | 0.82 (0.79-0.84) | 0.63 (0.60-0.67) | 0.59 (0.56-0.62) | 2.30 (2.22-2.38) |
| Interval | 18-23 | *ref* | *ref* | *ref* |  | *ref* | *ref* |
| <18 | | 0.87 (0.80-0.94) | 1.08 (0.98-1.19) | 1.12 (1.06-1.18) |  | 0.91 (0.83-0.99) | 0.92 (0.88-0.97) |
| >23 | | 1.20 (1.11-1.30) | 1.25 (1.14-1.38) | 1.15 (1.09-1.21) |  | 1.06 (0.97-1.15) | 0.98 (0.94-1.03) |
| NA | | 1.76 (1.64-1.89) | 2.03 (1.86-2.21) | 1.32 (1.26-1.39) |  | 1.58 (1.46-1.71) | 1.10 (1.05-1.15) |
| Outcome previous pregnancy | | 5.20 (4.76-5.68) | 8.98 (7.92-10.17) | 1.36 (1.22-1.52) | 1.49 (1.21-1.83) | 8.16 (7.55-8.81) | 11.05 (10.52-11.61) |
| Age*Assisted reproduction | ≤25 |  | *ref* |  |  |  |  |
| 25-30 | |  | 0.81 (0.46-1.41) |  |  |  |  |
| 30-35 | |  | 0.70 (0.41-1.20) |  |  |  |  |
| >35 | |  | 0.55 (0.32-0.94) |  |  |  |  |

Empty cells indicated the variable was not included in the final model for the outcome. Abbreviations: AGA, average for gestational age; AS_5min_, Apgar score 5 minutes after birth; BMI, body mass index; GDM, gestational diabetes; LGA, large for gestational age; NA, not available; PPI, proton pump inhibitors; SGA, small for gestational age.
