# Supplementary material for: Population-based cohort study: proton pump inhibitor use during pregnancy in Sweden and the risk of maternal and neonatal adverse events
Source: BMC Med. 2022 Dec 20;20:492. doi: 10.1186/s12916-022-02673-x (PMC9768950; doi:10.1186/s12916-022-02673-x)
Supplement: Supplementary file 4 — Additional file 4: Table A3. Associations between PPI exposure and maternal and neonatal health outcomes including only firstborns. Results were obtained by multiple logistic regression and expressed as odds ratios (OR) with 95% confidence interval (CI). Empty cells indicated the variable was not included in the final model for the outcome. Abbreviations: AGA, average for gestational age; AS5min, Apgar score 5 min after birth; BMI, body mass index; GDM, gestational diabetes; LGA, large for gestational age; NA, not available; PPI, proton pump inhibitors; SGA, small for gestational age. [file 12916_2022_2673_MOESM4_ESM.docx]

ADDITIONAL FILE 4: Table A3: Associations between PPI exposure and maternal and neonatal health outcomes including only firstborns. Results were obtained by multiple logistic regression and expressed as odds ratios (OR) with 95% confidence interval (CI).

|  | | Pre-eclampsia | GDM | Preterm | AS_5min_ <7 | SGA | LGA |
| --- | --- | --- | --- | --- | --- | --- | --- |
| PPI | | 1.21 (1.09-1.34) | 1.38 (1.16-1.63) | 1.17 (1.06-1.29) | 1.04 (0.86-1.24) | 1.34 (1.18-1.51) | 0.89 (0.74-1.06) |
| Age | ≤25 | *ref* | *ref* | *ref* |  | *ref* | *ref* |
| 25-30 | | 0.97 (0.93-1.00) | 1.20 (1.12-1.29) | 1.03 (0.99-1.06) |  | 0.96 (0.92-1.00) | 0.92 (0.87-0.97) |
| 30-35 | | 1.03 (0.99-1.07) | 1.38 (1.28-1.49) | 1.03 (0.99-1.06) |  | 1.12 (1.08-1.18) | 0.85 (0.80-0.90) |
| >35 | | 1.25 (1.19-1.31) | 1.90 (1.74-2.07) | 1.16 (1.10-1.21) |  | 1.49 (1.41-1.58) | 0.86 (0.79-0.93) |
| BMI | Normal | *ref* | *ref* | *ref* | *ref* | *ref* | *ref* |
| Under | | 0.78 (0.74-0.83) | 0.85 (0.75-0.96) | 1.09 (1.05-1.14) | 0.92 (0.85-1.00) | 1.49 (1.42-1.56) | 0.45 (0.40-0.50) |
| Over | | 1.63 (1.57-1.68) | 2.02 (1.89-2.17) | 1.05 (1.01-1.08) | 1.15 (1.09-1.22) | 0.88 (0.84-0.91) | 2.02 (1.92-2.13) |
| Obese | | 2.90 (2.79-3.01) | 5.02 (4.69-5.37) | 1.10 (1.05-1.14) | 1.52 (1.42-1.63) | 0.90 (0.85-0.95) | 3.13 (2.95-3.31) |
| NA | | 1.34 (1.27-1.41) | 1.32 (1.17-1.49) | 1.78 (1.71-1.86) | 1.39 (1.28-1.52) | 1.08 (1.01-1.15) | 1.49 (1.37-1.62) |
| Tobacco consumption | | 0.81 (0.76-0.85) | 1.17 (1.06-1.29) | 1.18 (1.12-1.24) |  | 1.86 (1.77-1.96) | 0.74 (0.68-0.81) |
| Comorbidities | | 1.95 (1.87-2.04) | 4.70 (4.40-5.03) | 1.63 (1.56-1.71) |  | 1.00 (0.93-1.07) | 3.24 (3.06-3.44) |
| Pre-eclampsia | |  | 1.62 (1.48-1.76) | 4.34 (4.19-4.50) | 1.15 (1.06-1.24) | 4.03 (3.85-4.22) | 1.52 (1.41-1.63) |
| Other drugs | | 1.15 (1.12-1.19) | 1.15 (1.09-1.22) | 1.07 (1.04-1.10) | 1.06 (1.01-1.12) | 0.99 (0.96-1.03) | 1.19 (1.13-1.24) |
| Assisted reproduction | | 1.10 (1.04-1.17) |  | 1.24 (1.17-1.31) |  | 0.99 (0.92-1.07) | 1.29 (1.18-1.41) |
| Mode of delivery | |  |  |  | 2.77 (2.64-2.91) |  |  |
| Preterm birth | |  |  |  | 4.14 (3.90-4.39) | 3.53 (3.38-3.68) | 1.58 (1.47-1.70) |
|  | |  |  |  |  |  |  |
|  | |  |  |  |  |  |  |
| Birthweight | AGA |  |  |  | *ref* |  |  |
| SGA | |  |  |  | 1.53 (1.40-1.67) |  |  |
| LGA | |  |  |  | 1.27 (1.12-1.45) |  |  |

Empty cells indicated the variable was not included in the final model for the outcome.

Abbreviations: AGA, average for gestational age; AS_5min_, Apgar score 5 minutes after birth; BMI, body mass index; GDM, gestational diabetes; LGA, large for gestational age; NA, not available; PPI, proton pump inhibitors; SGA, small for gestational age.
